# Parasitosis of the vertebral canal mimicking lumbar intervertebral disc herniation: a case report

**DOI:** 10.1186/s12891-020-03870-6

**Published:** 2021-01-05

**Authors:** Marcin Tyrakowski, Magdalena Kwiatkowska, Maria Czubak-Wrzosek, Jarosław Czubak

**Affiliations:** 1 Department of Spine Disorders and Orthopedics, Centre of Postgraduate Medical Education in Warsaw, Konarskiego 13, 05-400 Otwock, Poland; 2 Department of Orthopedics, Pediatric Orthopedics and Traumatology, Centre of Postgraduate Medical Education in Warsaw, Konarskiego 13, 05-400 Otwock, Poland

**Keywords:** Dracunculiasis, Spinal parasite, Parasitosis of vertebral canal

## Abstract

**Study design:**

Case report.

**Background:**

It is a case of dracunculiasis of the spine mimicking lumbar intervertebral disc herniation.

**Case presentation:**

A 57 year-old Caucasian male was admitted to the hospital because of the left L5 radiculopathy lasting for 2 months. The pain in the left lower limb was associated with muscle weakness on dorsal flexion of the foot, paresthesia of the dorsal aspect of the foot and tingling in the big toe. Neurological examination revealed: muscle weakness on dorsal flexion of the foot, impaired light touch and pin prick test on the dorsal aspect of the foot and positive Lasègue’s sign. Magnetic resonance imaging (MRI) examination revealed L4-L5 intervertebral disc herniation with sequester compressing the left L5 nerve root. The open L4-L5 left side discectomy was performed. During the sequester evacuation 3 pieces of nematodes were removed and preserved in 10% of formaldehyde solution. After the surgery the patient was pain free with normal neurological examination. The diagnosis of dracunculiasis was based on the morphology of the nematode and on exclusion of the other parasites. DM infestation could not be confirmed with molecular testing that was impaired by the formaldehyde.

**Conclusions:**

Parasite infestation should be considered even in cases with obvious MRI of lumbar intervertebral disc herniation. If a nematode was found accidentally during any surgery it should be preserved in a 0.9% saline, not in formaldehyde, not to disturb the molecular tests.

## Background

Dracunculiasis, also known as guinea worm disease, is caused by a nematode worm, *Dracunculus medinensis* (DM). Dracunculiasis is transmitted to humans via drinking water containing copepods infected with larvae of DM. After being ingested the copepods release DM larvae that penetrate the host’s stomach, intestinal wall, and enter the abdominal cavity or retroperitoneal space where they grow. Mature DM females grow to a length of 100 cm, they penetrate deeply into the connective tissue adjoining long bones or joints and migrate typically within the subcutaneous tissue to the skin, mostly of the lower extremities. About 1 year after the infestation the female worm induces a painful blister formation on the skin. When the blister ruptures, the larvae are released by the adult female worm on contact with water and are subsequently ingested by copepods [1–4].

Unusual locations (besides the skin) of the mature DM in the human body have been described and involve the pancreas, lung, periorbital tissue, testes, pericardium and the spinal cord, radio-ulnar joint or Bartholin gland [2, 5–7].

The aim of this study was to present a case of dracunculiasis of the vertebral canal that was mimicking, both clinically and radiographically, lumbar intervertebral disc herniation.

## Case presentations

A 57 year-old Caucasian male was admitted to the hospital because of severe left L5 radiculopathy. The pain in the left lower extremity started 2 months prior to admission and was associated with severe low back pain that resolved spontaneously within 3 weeks. The patient described the pain as dull, radiating to his left buttock, posterior thigh, antero-lateral shin and dorsum of his foot (Visual Analog Scale for the leg pain (VAS leg) of 7 out of 10 points). The pain was associated with muscle weakness on dorsal flexion of the left foot, paresthesia of the dorsal aspect of the left foot and tingling in the left big toe.

The patient was in good medical condition, well-nourished (Body Mass Index of 31.3 kg/m^2^) and immunocompetent. He had a 10 year history of well-controlled blood hypertension and was allergic to bee stings. He denied any parasitic infections that he was aware of. He owned a dog that was living in the house and was regularly vaccinated and dewormed. No contact with wild animals was noted. Social history was remarkable for frequent travelling to Asia (Burma, Cambodia, Indonesia, Laos, Malaysia, Singapore, Thailand, Vietnam) and Africa (Nigeria) between 1994 and 2014 (during 21 years before the onset of the symptoms).

On physical examination the patient was alert and cooperative. His neurological examination was positive for muscle weakness on dorsal flexion of the left foot (4 out of 5 points according to Lovett’s scale). The light touch and pin prick test were also impaired on the dorsal aspect of the left foot. Lasègue’s sign was positive on 70 degrees flexion on the left side and negative on the right. No other abnormalities were detected.

**Fig. 1:**
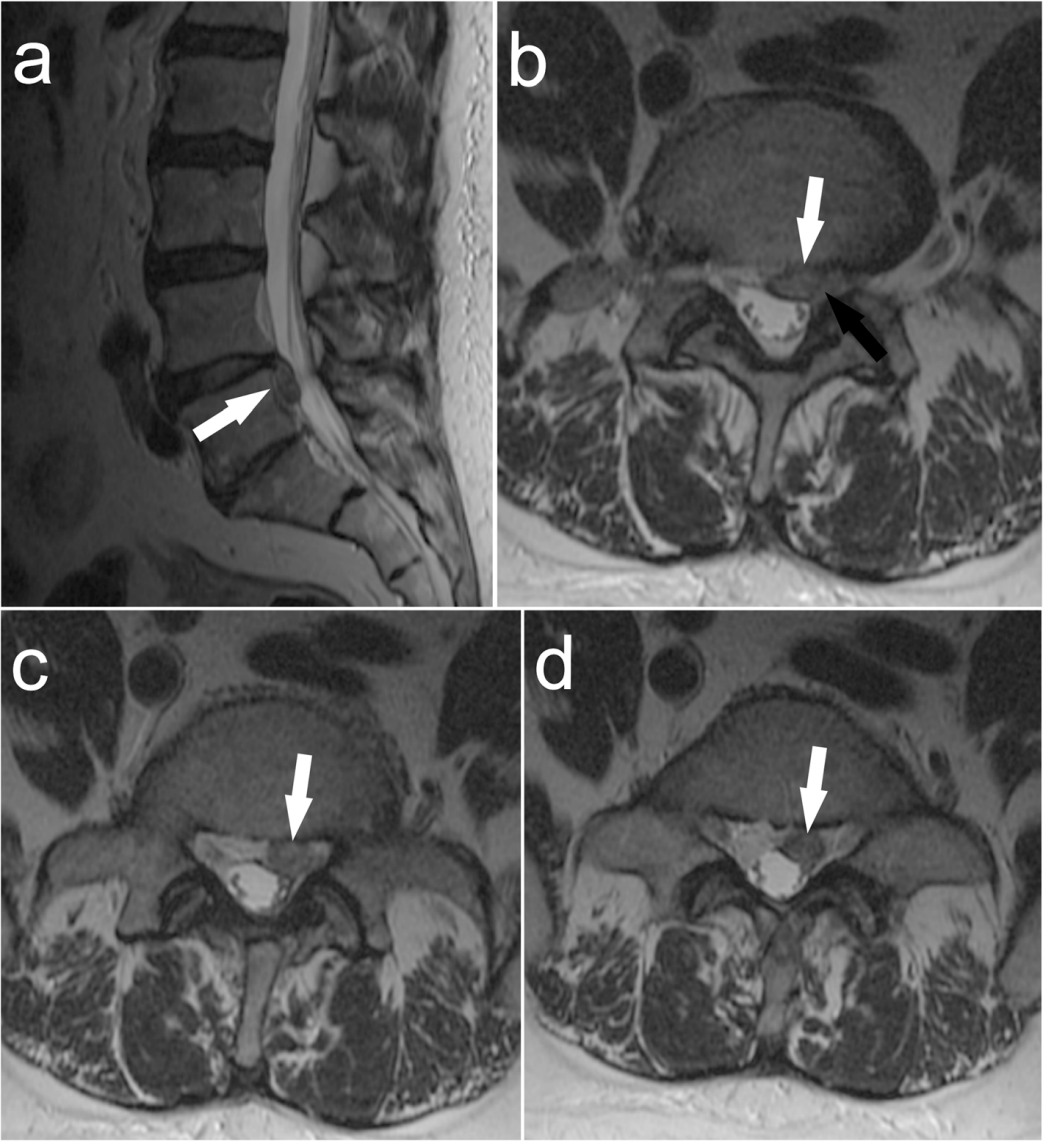
Magnetic resonance imaging (MRI) presenting parasitosis of the spinal canal at L4-L5 level (white arrow) with compression of the left L5 nerve root (black arrow): **a** – sagittal view; **b** – axial view at L4-L5 level; **c** and **d** - axial view at L5 level

Magnetic resonance imaging (MRI) examination of the lumbosacral spine revealed the L4-L5 intervertebral disc herniation with sequester compressing the left L5 nerve root within the left lateral recess of the spinal canal (Fig. 1). No abnormalities were found in the preoperative blood tests or urinalysis, with WBC count of 4,9 K/uL erythrocyte sedimentation rate of 8 mm/h and serum C-reactive protein concentration of 0,5 mg/L).

**Fig. 2:**
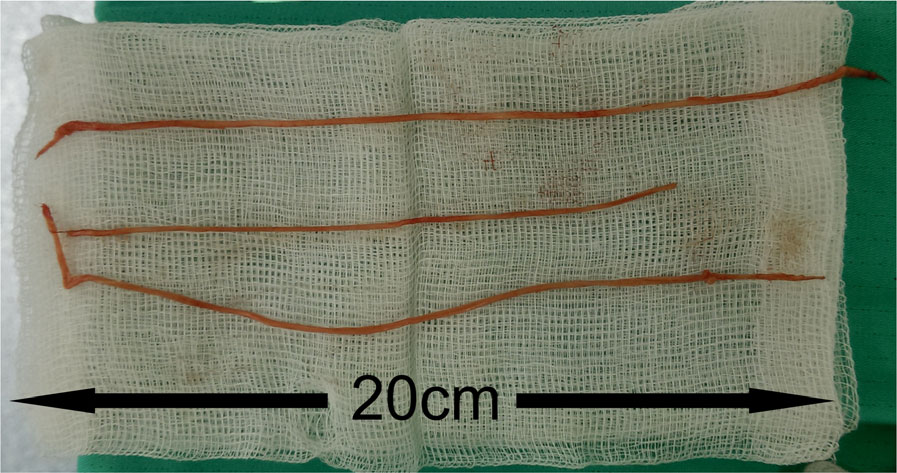
Intraoperative picture of the nematode worm that was removed from the bulge located on the anterior wall of the spinal canal and compressing the left L5 nerve root

**Fig. 3:**
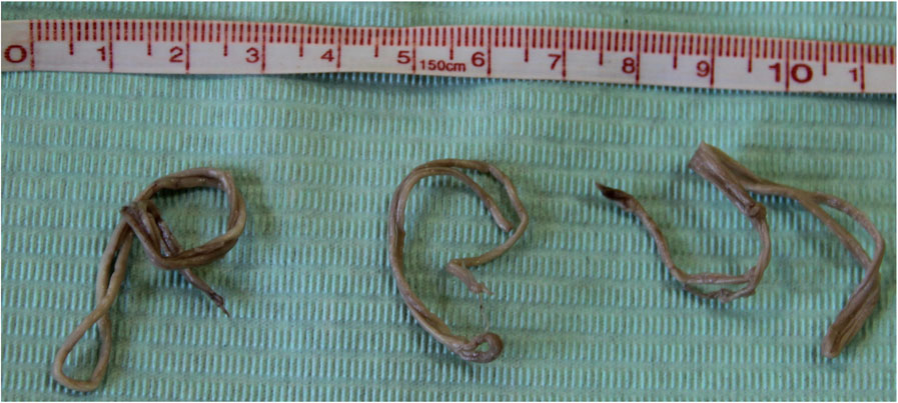
The nematode worm after preservation in 10% of formaldehyde solution

The patient was scheduled for an elective open left L4-L5 discectomy. During the sequester evacuation the surgeon removed 3 pieces of nematodes (length about 22 cm, 20 cm and 15 cm, respectively**)** that turned out to constitute the potential sequester compressing the L5 left nerve root (Fig. 2). The round worms were preserved in 10% formaldehyde and sent to the Department of Parasitology (Fig. 3). The preoperative, intraoperative and postoperative management was standard for an elective open discectomy, with antibiotic prophylaxis (2 g of Cefazolin iv 30 min before surgery) and irrigation with saline solution intraoperatively.

On postoperative day 1 the patient was pain free, besides a mild discomfort in the lumbar region. Lasègue’s sign was negative on both sides. Sensation deficits or muscle weakness were not detected. Slight tingling in the left big toe was still present. In accordance with our post-discectomy protocol, patient ambulation was started on postoperative day 1.

The following additional tests were performed: MRI with contrast (Gadolinium) of the head and cervical and thoracic spine as well as computed tomography (CT) with contrast of the chest and abdomen, but revealed no other entities of parasitosis. Total serum immunoglobulin E concentration was within the normal values. No microfilaria or elevated eosinophil count was found in the blood smear.

The serological ELISA tests for toxocarosis and filariasis were negative. The diagnosis of dracunculiasis was based on the morphology of the nematode. DM infestation could not be confirmed with the molecular testing that was impaired by the formaldehyde used for preservation of the worm.

No other treatment for the patient was administered, according to the recommendations of the Department of Parasitology.

After 3 years the patient was admitted to the spine department again because of numbness of both lower extremities. The numbness started a few months prior to admission and was associated with mild intermittent low back pain. Apart from light touch impairment on the dorsal aspect of the left foot, no other abnormalities were detected on neurological examination.

The MRI examination revealed a degenerative disease of the lumbar spine with mild spinal canal stenosis on L3-L4 level. No sign of recurrence of dracunculiasis was observed. The patient was scheduled for an epidural steroid injection. After the procedure all symptoms resolved.

## Discussion and conclusions

Parasitic infection of the spine is a rare condition. The most common parasites affecting the spinal canal include cysticercosis, schistosomiasis, toxoplasmosis and echinococcal disease, and are much more frequent in the developing countries of Asia and Africa [8]. The symptoms are caused by spinal cord compression or bone destruction of the vertebrae and are easy to confuse with other spine diseases such as tuberculosis, pyogenic infection, tumor or even intervertebral disc herniation. The diagnosis of spinal parasitosis is usually based on radiological findings and significant clinical history. Nowadays the gold standard for diagnosis of spinal parasitosis is MRI. Basing on radiological image one can differentiate various spinal parasitic infections. Neurocysticercosis can affect very diverse locations including the vertebrae, spinal cord, epidural, subdural or subarachnoid space, whereas neuroschistosomiasis usually forms an intramedullary granuloma. A typical manifestation of spinal cord toxoplasmosis is vacuolar myelopathy, with intramedullary ring-enhancing lesions. The last of the most common forms of spinal parasitosis, echinococcal disease, can cause cystic lesions in the vertebral bodies, bone lysis, spondylitis or spinal cord cysts [8].

This is the first report of dracunculiasis of the vertebral canal since the 1980s, when several cases were described [9–11] and the first ever described case where modern radiological imaging, such as computed tomography and magnetic resonance imaging, has been used.

Commonly, the first clinical manifestation of CNS dracunculiasis occurs 12 months after inoculation or skin manifestation. There are 3 types of neurological disorders caused by guinea worm: diffuse encephalopathy with intracerebral granulomas, intracranial hypertension due to blockage of CSF flow in the ventricular system by a granuloma and according to literature the least common, epiduritis, caused by adult worm migration [9]. The route of migration of dracunculus medinensis to the extradural space is probably from the retroperitoneal space through one of the intervertebral foramina [12].

Most of the previously reported cases were located in the epidural space of the cervical and upper thoracic spine, although the literature search revealed one case of lumbar spine infestation [9–12]. In this case the parasite caused extradural compression in the lumbar region with osteomyelitis of L5, that lead to a collapse of the vertebra. According to the authors an injured or lacerated guinea worm can cause a severe local inflammatory response [12].

Majority of patients presented symptoms of severe spinal canal stenosis including tetra- and paraparesis. Radiological findings include calcifications in the spinal canal at the level of compression on radiographs and complete or incomplete epidural block in myelography [9–11]. The laboratory tests were mostly negative, with a slightly elevated level of eosinophils in blood smear in some cases [11].

Intraoperatively, after a laminectomy and inspection of the vertebral canal, a granuloma with calcified nematode was usually found; however, in one case the surgeon evacuated from an epidural lesion a 60 cm long living parasite [10].

Literature does not describe an optimal surgical protocol for the treatment of dracunculiasis in unusual locations. The main treatment for common skin location is extraction of the parasite by gentle traction and winding around a stick, which can last from hours to months. The antihelminthic drugs such as niridazole, thiabendazole, or metronidazole can reduce the acute tissue reaction and help to extract the worm more easily [4].

Based on observations, two different types of evolution of spinal dracunculiasis have been distinguished:

- acute form – where neurological deficits develop in hours, and are preceded by 2–4 months of back pain, with poor results of treatment
- subacute form – with months or weeks of back and radicular pain, with good results of treatment [11].

Although it is a rare condition, dracunculiasis should be taken into consideration even in cases with obvious lumbar intervertebral disc herniation seen on MRI, especially in patients travelling to the endemic areas. If a nematode is found accidentally during any surgery, it should be preserved in a 0.9% saline, not formaldehyde, to give valid results in molecular tests.

## Data Availability

All data generated or analysed during this study are included in this published article.

## Abbreviations

DM: *Dracunculus medinensis*
VAS: Visual Analog Scale
MRI: Magnetic resonance imaging
CT: Computed tomography
CNS: Central Nervous System
CSF: Cerebrospinal Fluid
